# Evaluation of malaria rapid diagnostic test (RDT) use by community health workers: a longitudinal study in western Kenya

**DOI:** 10.1186/s12936-018-2358-6

**Published:** 2018-05-18

**Authors:** Matthew R. Boyce, Diana Menya, Elizabeth L. Turner, Jeremiah Laktabai, Wendy Prudhomme-O’Meara

**Affiliations:** 1 Duke Global Health Institute, Duke University, Durham, NC USA; 2 Department of Epidemiology and Biostatistics, Moi University School of Public Health, College of Health Sciences, Eldoret, Kenya; 3 Department of Biostatistics and Bioinformatics, Duke University, Durham, NC USA; 4 Department of Family Medicine, Moi University School of Medicine, Eldoret, Kenya; 5 Duke University Medical Center, Durham, NC USA

**Keywords:** Community health worker, Malaria, Rapid diagnostic test, RDT, Kenya, Sub-Saharan Africa, Longitudinal

## Abstract

**Background:**

Malaria rapid diagnostic tests (RDTs) are a simple, point-of-care technology that can improve the diagnosis and subsequent treatment of malaria. They are an increasingly common diagnostic tool, but concerns remain about their use by community health workers (CHWs). These concerns regard the long-term trends relating to infection prevention measures, the interpretation of test results and adherence to treatment protocols. This study assessed whether CHWs maintained their competency at conducting RDTs over a 12-month timeframe, and if this competency varied with specific CHW characteristics.

**Methods:**

From June to September, 2015, CHWs (*n *= 271) were trained to conduct RDTs using a 3-day validated curriculum and a baseline assessment was completed. Between June and August, 2016, CHWs (*n *= 105) were randomly selected and recruited for follow-up assessments using a 20-step checklist that classified steps as relating to safety, accuracy, and treatment; 103 CHWs participated in follow-up assessments. Poisson regressions were used to test for associations between error count data at follow-up and Poisson regression models fit using generalized estimating equations were used to compare data across time-points.

**Results:**

At both baseline and follow-up observations, at least 80% of CHWs correctly completed 17 of the 20 steps. CHWs being 50 years of age or older was associated with increased total errors and safety errors at baseline and follow-up. At follow-up, prior experience conducting RDTs was associated with fewer errors. Performance, as it related to the correct completion of all checklist steps and safety steps, did not decline over the 12 months and performance of accuracy steps improved (mean error ratio: 0.51; 95% CI 0.40–0.63). Visual interpretation of RDT results yielded a CHW sensitivity of 92.0% and a specificity of 97.3% when compared to interpretation by the research team. None of the characteristics investigated was found to be significantly associated with RDT interpretation.

**Conclusions:**

With training, most CHWs performing RDTs maintain diagnostic testing competency over at least 12 months. CHWs generally perform RDTs safely and accurately interpret results. Younger age and prior experiences with RDTs were associated with better testing performance. Future research should investigate the mode by which CHW characteristics impact RDT procedures.

**Electronic supplementary material:**

The online version of this article (10.1186/s12936-018-2358-6) contains supplementary material, which is available to authorized users.

## Background

Malaria remains a leading public health problem, causing high levels of morbidity and mortality worldwide, particularly in sub-Saharan Africa, where an estimated 90% of all malaria deaths occur [1]. Previously, malaria case management relied on the presumptive treatment of febrile illness with anti-malarial drugs [2]. However, malaria treatment policy has changed and presumptive treatment no longer represents the recommended approach for malaria case management. In 2010, the World Health Organization revised their recommendations to require parasitological confirmation of malaria infection prior to treatment, also known as the ‘test-and-treat’ strategy [3]. This change was precipitated by a declining prevalence of malaria in sub-Saharan Africa [4–6], evidence suggesting that malaria only causes a proportion of all febrile illness in malaria-endemic regions [7–9], concerns surrounding anti-malarial drug resistance [10, 11], and improvements in diagnostic technologies [12, 13]. The confirmation of parasitological infection is also important for the management of non-malaria febrile illnesses [2].

Malaria rapid diagnostic tests (RDTs) are immuno-chromatographic tests that detect the presence of malaria antigens released from parasitized red blood cells. RDTs require that healthcare workers obtain a blood sample from a patient using a lancet, place the sample and a buffer solution in a test cassette, and interpret the results. The tests do not require electricity or specialized equipment and return results within 30 min [14, 15]. Systematic reviews have shown that RDTs are accurate [13] and cost effective [16, 17]. The simplicity of these tests eliminates the need for high levels of technical expertise and allows for them to be used by a wide range of personnel, which may dramatically reduce the presumptive use of anti-malarial drugs [18, 19].

Community-based health interventions are becoming increasingly prevalent, especially in resource-constrained settings [20]. They can improve access to health services in medically underserved areas and reduce the burden felt by health systems by task shifting [21]. In Kenya, these interventions are most often implemented by community health workers (CHWs), a volunteer, non-salaried workforce involved in health campaigns, health promotion and referral. CHWs are an integral part of the 2006 Kenya Community Health Strategy and are linked to local health facilities through a government employed community health extension worker (CHEW) [22]. The current strategy for community case management of malaria pairs RDTs and artemisinin-combination therapy (ACT) in the event of a positive test result [23].

Previous work has demonstrated that CHWs can safely and accurately use RDTs after receiving training and instruction [17]. However, concerns remain pertaining to the long-term use of RDTs by CHWs, specifically how to monitor them over time to ensure the quality of testing is maintained. Concerns that have been most commonly noted in literature relate to infection prevention and control measures (e.g., blood safety), the ability to correctly interpret test results, and adherence to protocols [17, 24–28]. Furthermore, some speculate that overall performance and skill retention may be lower among those with little or no formal education [27]. Despite these reservations, large programmes relying on accurate malaria diagnosis by lay health persons continue to be implemented in many countries [29–31].

To address these concerns, an observational study was conducted amongst CHWs participating in a large malaria RDT programme in western Kenya [32]. The objective of the study was to determine whether CHWs maintain their competency at conducting RDTs over time and if performance differs across sociodemographic characteristics of the CHWs. Specifically, the study sought to (i) evaluate if the ability of CHWs to safely and correctly administer RDTs changes over time; (ii) examine which, if any, steps are performed incorrectly; (iii) determine if performance of the RDT procedure varies with selected characteristics; and, (iv) evaluate if CHWs demonstrate high levels of competence in interpreting malaria RDT results 12 months post-training.

## Methods

### Study context and site

This study was part of an ongoing, cluster-randomized, controlled trial to evaluate an intervention to improve targeting of anti-malarials [32]. The study ran from June 2015 to July 2016 in 16 communities in Bungoma East sub-county and Kiminini sub-county in western Kenya. Study areas are located in western Kenya roughly 50 km east of the Ugandan border. The sub-counties have a similar malaria burden, predominantly characterized by *Plasmodium falciparum* with perennial transmission patterns.

### Study procedures

#### Community health worker training

Community health workers affiliated with the Kenyan government, who were part of an ongoing, cluster-randomized, controlled trial, were eligible for the current study [32]; 32 clusters of communities were enrolled, and 16 were randomly selected for inclusion in the trial. CHWs from the selected communities, who had previously received training from the Kenyan Ministry of Health (MOH), received additional RDT training between June and September, 2015 using a validated 3-day curriculum derived from materials from the Kenyan MOH in conjunction with skills-oriented training sessions. Training personnel included researchers from the study team, nurses, public health officers, members of the sub-county health management team, and peer mentors with extensive experience administering RDTs. A total of 271 CHWs successfully completed the training by demonstrating competence in completing the RDT procedure and interpretation of test results, and were equipped with a supply of RDTs (CareStart Malaria HRP2 (Pf); AccessBio Inc, USA), including all the supplies required to perform the tests. The supplies provided included a thermometer, placemat, pen, gloves, lancet, pipette, alcohol swab, cotton, buffer solution, RDTs, sharps box, non-sharps disposal bag, and a water-proof transport bag, allowing them to perform RDTs independently in their own community.

Community health workers offered free RDT testing to febrile individuals or those presenting with malaria-like symptoms. Conditional on a positive test result, CHWs were trained to provide individuals with the positive test with a voucher with the testing details allowing for the purchase of quality-assured ACT medicines at a reduced, fixed price at a participating pharmacy. Pharmacies would only dispense ACT to individuals presenting both the positive test and accompanying voucher with matching details. Those with negative test results or severe illness (of any origin) were referred to a health facility for further testing. The sub-county health team and study team met with groups of CHWs once per month to discuss challenges, replenish supplies, and to spot check RDT storage. RDT procedures were not practiced or observed at monthly supervision meetings.

A sample of 100 randomly-selected CHWs was selected into groups of 10 and received additional training on how to use a small, automated, battery-operated device known as a Deki reader (DR) (Fio Corporation, Toronto, Canada) [33]. These devices were developed to improve the interpretation of RDTs through image analysis software [34]. Briefly, CHWs were instructed to use DRs on 10 successive patients over the span of about 1 month between July 2015 and April 2016. For a DR-evaluated RDT, CHWs recorded a unique study identifier on the RDT cassette, placed it in the DR for a photograph, removed the cassette and performed the RDT. After performing the RDT, CHWs inserted the cassette into the DR once again and used the machine to record his or her interpretation. The DR took a second photograph of the final RDT for automated interpretation of the results. The CHWs interpretation of RDT results was compared with the Deki reader’s automatic interpretation. When all 10 CHWs in the group had conducted at least 10 DR-RDT tests, or the time limit with the DR had been reached, the DRs were rotated to the next group of 10 randomly selected CHWs. This study is described in detail elsewhere [33]. Financial constraints prevented all CHWs from receiving Deki reader training.

#### Baseline data collection

Baseline data were collected from all CHWs who attended the 2015 RDT training. Data were collected using a standardized RDT observation checklist at the training that was based on one validated through previous work [25]. The checklist divided the RDT procedure into 20 steps in three domains: safety (five steps), accuracy (seven steps), and procedural (eight steps) (Table 1). A member of the training team watched CHWs conduct one RDT procedure on the final day of training, noting whether the CHW performed each step correctly, incorrectly, or not at all. These tests were performed on volunteers of unknown malaria infection status and diagnostic results were confirmed by the training team member. Anti-malarials were provided to any individual testing positive for malaria. Prior experiences with RDTs, time employed as a CHW, prior training in malaria case management, as well as sociodemographic characteristics (gender, age, education level, marital status, formal employment status) were recorded using standardized survey forms.

**Table 1:** Observation checklist and step-by-step performance of community health workers

No.	Task category	Task	% of CHWs completing each step correctly	
			Baseline (*n *= 90)	12 months (*n *= 103)
1.	P	Assembles necessary materials	92.2	89.3
2.	A	Read RDT expiration date	53.3	52.4
3.	P	Remove contents of test packet	98.9	99.0
4.	P	Write patient’s name on cassette	92.2	94.2
5.	P	Identify patient’s details and date on the RDT cassette	87.8	92.2
6.	P	Explain procedure to patient	66.7	73.8
7.	S	Wear gloves	97.8	95.2
8.	P	Select 4th finger from the thumb of the left hand for blood collection	93.3	86.4
9.	S	Clean finger with alcohol swab and allow it to dry	95.6	89.3
10.	S	Prick finger firmly with sterile lancet	96.7	94.2
11.	S	Discard lancet in sharps bin immediately after pricking finger	95.6	95.2
12.	P	Do not squeeze finger excessively	84.1	88.3
13.	A	Collect an adequate volume of blood with pipette	78.9	87.4
14.	A	Dispense blood in correct well	97.8	98.1
15.	S	Discards the pipette in the sharps box	98.8	92.2
16.	P	Dispose of gloves and cotton wool in non-sharps container	94.4	70.9
17.	A	Dispense correct volume of buffer	97.8	97.1
18.	A	Wait for 20 min	96.6	96.1
19.	A	Read results correctly	90.4	99.0
20.	A	Verify internal test control	97.6	88.4

Time-point	Summary statistics (range)	Mean (SD)	Median (IQR)
Baseline	Total steps correct (0–20)	17.80 (1.65)	18 (17–19)
	Safety steps correct (0–5)	4.80 (0.48)	5 (5)
	Accuracy steps correct (0–7)	5.39 (0.98)	6 (5–6)
12 months	Total steps correct (0–20)	17.78 (1.89)	18 (17–20)
	Safety steps correct (0–5)	4.66 (0.60)	5 (4–5)
	Accuracy steps correct (0–7)	6.18 (0.81)	6 (6–7)

P steps relate to procedural aspects, S steps relate to test safety and A steps relate to test accuracy. Of the 103 participating CHWs, baseline checklists for 13 CHWs were unable to be located and were not included in baseline data analysis; 90 baseline observations exist, although some checklists (*n *= 14) had incomplete observation data resulting missing scores for no more than two steps. For additional information please see Additional file 1

#### Recruitment of participants for follow-up observations

In June 2016, CHWs who had completed RDT training were recruited for a follow-up assessment. Test quality, defined as a relative per cent change in the completion of a step, may be expected to change from between 4 and 20% over a 12-month period [25]. Assuming that at the completion of their training CHWs performed all 20 steps correctly, the study team was interested in detecting reduction to 90% in the proportion of CHWs performing all 20 steps correctly at follow-up. To do so, a minimum of 90 CHWs were required to estimate a 95% confidence interval for that proportion with a margin of error of 6%. Based on this sample size calculation, a minimum of 90 CHWs were targeted at follow-up, and a total of 105 CHWs were invited to allow for some loss to follow-up. The sample was randomly selected from a complete list of CHWs using Microsoft Excel (Redmon, WA, USA). All CHWs were randomly assigned a number, sorted from lowest to highest, and the first 105 CHWs were invited to participate in the study. At follow-up, 103 CHWs were observed. One CHW who did not participate because she was on maternity leave; another CHW had recently deceased. Baseline data RDT checklist data could not be located for 13 participants (Additional file 1). Sensitivity analyses were conducted to investigate whether these populations differed significantly.

#### Follow-up data collection

Initial observations and follow-up observations were designed to be as similar as possible to allow for the comparison of observations. Two of the training personnel acted as observers for follow-up collection and received 1 day of training including instruction on how to minimize observer-induced bias.

In an effort to assess CHW performance as close as possible to real-life conditions, CHWs were instructed to report to local health facilities for a 1-day malaria testing exercise. The study team communicated with health facility staff to request that febrile patients be sent to the CHWs for malaria testing, rather than to the clinical laboratory as usual. Using the same checklist as baseline, an observer would watch CHWs conduct one RDT procedure, noting whether the CHW performed each step correctly, incorrectly, or not at all. Observers did not interrupt the CHW except if one of the five safety steps (steps 7, 9, 10, 11, 15) or two accuracy steps (steps 18 or 19) were performed incorrectly. These steps represented an ethical dilemma, as failure to correctly complete them could compromise patient safety or accurate test results.

Following testing, CHWs referred patients back to the health facility with an encounter form where the date, patient’s name, age, temperature at the time of testing, and malaria RDT result were recorded to allow the patient to receive appropriate follow-up treatment. Patients were excluded from the study and referred back to the health facility staff if they were under 1 year old, pregnant, or displaying severe illness. On six occasions, no febrile patient was available for testing. In these instances, volunteers from the research team were recruited, asked to provide informed verbal consent, and used to assess CHW RDT performance.

Community health workers were also requested to participate in an interpretation assessment at follow-up. This assessment involved the interpretation of ten RDTs. The RDTs used in the assessment had been previously conducted and collected by the study team prior to follow-up assessments. The study team selected RDTs to represent positive, negative and invalid test results. To assess CHW test result interpretation, each CHW was asked to interpret each of the tests as positive, negative or invalid. The gold standard for this assessment was the interpretation of the research team.

### Data analysis

Checklists and interpretation assessments were collected on paper forms and input to Microsoft Excel. Data were reviewed for consistency and validity to ensure data accuracy and completeness prior to beginning analysis. Data were analysed using STATA version 14.0 (College Station, TX, USA). The percentage of steps performed correctly at baseline and 12 months, as well as counts of errors in total steps, safety steps, and accuracy steps were calculated for both time-points. Univariable and multivariable Poisson regressions were fit to test for associations between error count data at the 12-month time-point and pre-specified covariates (gender, age, education level, marital status, formal employment status, prior RDT experiences, prior CHW work experience, prior training in malaria case management, DR experiences).

The longitudinal analysis included Poisson regression models fit using generalized estimating equations (GEE) with an exchangeable correlation matrix to account for correlation between test scores from the same CHW collected at 2 time-points [35]. Selected pre-specified covariates (gender, age, education level, prior RDT experiences, prior CHW work experience, prior training in malaria case management, DR experiences) were included in the analysis based on their hypothesized relationship to the outcome. This analysis was used to identify factors associated with RDT performance at both time-points (baseline or 12-months).

Sensitivity and specificity of test interpretation were calculated for all tests. Multivariable logistic regression analysis was used to examine the relationship between committing at least one error in the RDT interpretation assessment and selected covariates. Statistical significance for all analyses was defined as *P *< 0.05.

### Ethical approval

Verbal informed consent was obtained from both CHWs and patients, or caregivers in the event of a patient under the age of 18 years. The study protocol was approved by the Duke University Institutional Review Board (Durham, NC) and the Moi University Institutional Research and Ethics Committee (Eldoret, Kenya).

## Results

### Participant demographics

Sociodemographic characteristics of the 103 participating CHWs are presented in Table 2. They were 43.6 years old on average and most were women (68.9%), were married (82.5%), and a majority (58.3%) had completed secondary school or higher education. Half (51.5%) had prior experience working as a CHW, one-third (32.2%) had received prior training in malaria case management and several (17.5%) had prior experiences with malaria RDTs. Sensitivity analyses showed that no statistically significant differences existed between the populations.

**Table 2:** Characteristics of participating community health workers (*n *= 103)

Characteristic	n (% or range)
Gender	
Male	32 (31.1)
Female	71 (68.9)
Mean age (years)	43.6 (20–69)
≤ 39	36 (35.0)
40–49	42 (40.8)
≥ 50	25 (24.3)
Education (highest level attained)	
Primary or less	43 (41.7)
Secondary or greater	60 (58.3)
Married	85 (82.5)
Formally employed	16 (15.5)
Had prior CHW work experience	53 (51.5)
Mean work experience (years)	4.1 (0–6)
Had prior malaria treatment experience	33 (32.2)
Had prior malaria RDT experience	18 (17.5)

Characteristics were recorded at baseline observations

### Baseline RDT performance

At baseline, each step was carried out correctly by greater than 80% of participants, except for steps 2, 6, and 13 (Table 1). None of these steps were safety steps, although steps 2 and 13 were accuracy steps. The median number of total steps completed correctly was 18 (IQR: 17–19), the median number of safety steps correctly completed correctly was 5 (IQR: 5), and the median number of accuracy steps correctly completed was 6 (IQR: 5–6). The number of errors observed ranged from 0 to 4 errors per CHW.

All safety steps were correctly completed by at least 95% of CHWs at baseline observations. At baseline observations, greater than 80% of CHWs completed 5 of the 7 accuracy steps. The steps most commonly performed incorrectly by CHWs were steps 2 and 13.

### Follow-up RDT performance

At follow-up, each step was carried out correctly by > 80% of CHWs except for steps 2, 6 and 16 (Table 1). The median number of total steps completed correctly was 18 (IQR: 17–20), the median number of safety steps completed correctly was 5 (IQR: 4–5), and the median number of accuracy steps correctly completed was 6 (IQR: 6–7). The number of errors observed at follow-up observations ranged from 0 to 11 errors per CHW. An additional file details the errors observed (Additional file 2).

At follow-up observations, greater than 90% of CHWs completed 4 of 5 safety steps. Only step 9, which entailed the cleaning of a patient’s finger, was completed incorrectly by greater than 10% of CHWs. The most common error relating to properly cleaning a patient’s finger with alcohol (step 9) was excessive wiping or scrubbing. One CHW attempted to conduct the RDT procedure without cleaning the patient’s finger with alcohol. On four occasions at follow-up observations, CHWs opened the lancet and placed it down before pricking the patient’s finger, compromising the sterility of the tool (step 10). The most common error regarding the disposal of used lancets was placing it near but not in the sharps box. Only once did a CHW dispose of a used lancet in the non-sharps container.

At follow-up observations, greater than 90% of CHWs completed 4 of the 7 accuracy steps, and greater than 80% of CHWs completed 6 of the 7 steps. The most frequent errors were for step 2, which was completed correctly in 52.4% of the observations. Other errors related to collecting an adequate volume of blood, which was completed correctly by 87.4% of CHWs, and verifying the internal test control which was completed correctly by 88.4% of CHWs.

Poisson regressions modelling the outcome of total error count at 12 months showed that CHW gender, prior RDT training, and age were associated with overall test performance (Table 3). The mean error ratio (MER) for gender was 0.70 (95% CI 0.52–0.96), which corresponds to the mean number of total errors for men being 30% (95% CI 4–48) lower when compared to women. The MER for CHWs 50 years of age or older compared to CHWs younger than 40 years of age was 1.69 (95% CI 1.23–2.31), corresponding to the mean number of total errors being 69% (95% CI 23–131) higher for CHWs aged 50 or older. The MER for prior RDT experience was 0.50 (95% CI 0.31–0.80), which corresponds to the number of total errors being 50% (95% CI 20–69) lower for those with RDT training prior this training program compared to those without prior training.

**Table 3:** Covariates associated with total error counts at follow-up observations using multivariable Poisson regression (*n *= 103)

Variable	MER (95% CI)	P value
Male	0.70 (0.52–0.96)	0.029*
Age (years)		
< 40	0 (referent)	–
40–49	0.78 (0.57–1.07)	0.181
≥ 50	1.69 (1.23–2.31)	0.001*
Education		
Primary or less	0 (referent)	–
Secondary or greater	0.91 (0.72–1.14)	0.395
Married	0.93 (0.67–1.30)	0.683
Formal employment	1.13 (0.78–1.64)	0.519
Had prior CHW experience	0.84 (0.65–1.08)	0.169
Had prior malaria experience	1.37 (0.98–1.91)	0.062
Had prior RDT experience	0.50 (0.31–0.80)	0.004*
Had Deki reader experience	0.90 (0.68–1.18)	0.435

Data are presented for 103 CHWs who were observed at follow-up observations. Mean error ratios (MER) were used to compare CHWs that belonged to categorically different covariate groups (e.g., formally employed vs no formal employment)

* Denotes statistically significant results (*P *< 0.05)

### RDT performance comparison

When compared to baseline observations, a higher percentage of CHWs were observed to correctly complete steps 5, 6, 13, and 19, but fewer CHWs correctly completed steps 8, 9, 15, 16, and 20 (Table 1).

Results from GEE modelling demonstrated that, on average, CHW performance did not differ significantly between baseline and 12 months post-training for all steps and safety steps. GEE modelling (Table 4) showed that the error rates for accuracy steps were significantly reduced by half at 12 months (MER = 0.51, 95% CI 0.40–0.63). Characteristics related to age were associated error rates at a statistically significant level in the GEE model that included both baseline and follow-up observations. Being 50 years of age or older was observed to be associated with a higher number of total errors (MER = 1.39, 95% CI 1.04–1.87) and safety errors (MER = 3.58, 95% CI 1.64–7.81).

**Table 4:** Covariates associated with error count data at two time-points using generalized estimating equation models (*n *= 103)

Variable	Total errors		Safety errors		Accuracy errors	
	MER (95% CI)	P value	MER (95% CI)	P value	MER (95% CI)	P value
Time						
Baseline	1.00 (referent)		1.00 (referent)		1.00 (referent)	
Follow-up	0.88 (0.72–1.07)	0.208	1.65 (0.96–2.82)	0.066	0.51 (0.40–0.63)	0.000*
Gender						
Female	1.00 (referent)		1.00 (referent)		1.00 (referent)	
Male	0.91 (0.70–1.18)	0.497	1.16 (0.64–2.10)	0.626	1.06 (0.82–1.36)	0.656
Age (years)						
≤ 39	1.00 (referent)		1.00 (referent)		1.00 (referent)	
40–49	0.91 (0.71–1.18)	0.486	1.37 (0.61–3.07)	0.440	0.87 (0.71–1.12)	0.308
≥ 50	1.39 (1.04–1.87)	0.025*	3.58 (1.64–7.81)	0.001*	1.24 (0.93–1.65)	0.144
Education						
Primary or less	1.00 (referent)		1.00 (referent)		1.00 (referent)	
Secondary or greater	1.09 (0.86–1.37)	0.475	1.17 (0.64–2.17)	0.599	1.21 (0.97–1.52)	0.097
CHW experience						
None	1.00 (referent)		1.00 (referent)		1.00 (referent)	
Experience	0.94 (0.75–1.17)	0.584	0.75 (0.41–1.37)	0.349	1.01 (0.82–1.26)	0.910
Malaria experience						
None	1.00 (referent)		1.00 (referent)		1.00 (referent)	
Experience	1.10 (0.87–1.40)	0.424	1.30 (0.66–2.53)	0.442	1.21 (0.95–1.53)	0.121
RDT experience						
None	1.00 (referent)		1.00 (referent)		1.00 (referent)	
Experience	0.95 (0.67–1.36)	0.787	1.21 (0.51–2.87)	0.660	1.14 (0.79–1.63)	0.488
Deki experience						
None	1.00 (referent)		1.00 (referent)		1.00 (referent)	
Experience	0.91 (0.71–1.16)	0.450	1.36 (0.75–2.46)	0.318	1.07 (0.85–1.36)	0.537

Mean error ratios (MER) were used to compare CHWs that belonged to categorically different covariate groups

*n *= 90 for baseline observations; *n *= 103 for follow-up observations

* Denotes statistically significant result (P < 0.05)

### Interpretation of RDT results

Community health worker interpretation of RDTs yielded high sensitivities and specificities when compared to research team interpretation. The overall sensitivity at follow-up observations was 92.0% and the overall specificity was 97.3% (Table 5). The interpretation test also included one invalid test cassette, which all CHWs (*n *= 103) correctly interpreted. The cassettes most frequently misinterpreted were 2 with faint-positive results. Most CHWs (*n *= 92) correctly interpreted at least one of these cassettes correctly, and 73.8% (*n *= 76) correctly interpreted both faint-positive cassettes; 30 CHWs committed at least one error and the number of errors committed ranged from 0 to 4.

**Table 5:** Correct interpretation of RDT results 12 months post-training for 103 CHWs each evaluating 10 tests

	True positive	True negative
CHW positive	474	11
CHW negative	41	401

	Sensitivity	Specificity
	92.04	97.33

Excludes one invalid test cassette, which 100% of CHWs (n = 103) correctly interpreted

Multivariable logistic regression (Table 6) showed that none of the covariates had a statistically significant impact on RDT interpretation. Use of the Deki reader appeared to reduce the odds of committing an interpretation error by nearly 65%, although this did not reach statistical significance at the 0.05-level (Adjusted OR 0.36, 95% CI 0.13–1.04; *P *= 0.06).

**Table 6:** Covariates associated with committing at least one RDT interpretation error using multivariable logistic regression (*n *= 103)

Variable	Odds ratio (95% CI)	P value
Male	1.00 (0.33–2.99)	0.998
Age (years)		
< 40	0 (referent)	–
40–49	0.90 (0.31–2.65)	0.851
≥ 50	2.16 (0.65–7.23)	0.207
Education		
Primary or less	0 (referent)	–
Secondary or greater	0.61 (0.27–1.38)	0.237
Married	0.64 (0.18–2.23)	0.488
Formal employment	1.92 (0.50–7.35)	0.340
Had prior CHW experience	0.62 (0.27–1.77)	0.443
Had prior malaria experience	1.78 (0.50–6.30)	0.370
Had prior RDT experience	1.15 (0.244–5.48)	0.854
Had Deki reader experience	0.36 (0.13–1.04)	0.060

## Discussion

Results of this study demonstrate that CHWs generally adhere to testing procedures, can safely and accurately perform RDTs, and interpret test results correctly 12 months after they receive training. This adds to the growing body of evidence that CHWs perform RDTs at an acceptable level [13], and that these skills are maintained over time [25]. Although CHWs correctly performed most steps in the testing procedure, this study revealed several specific aspects, explaining the procedure and results to the patient, interpreting faint-positive test results, and other steps relating to accuracy, that hold implications for monitoring and improving future RDT use by CHWs.

Regarding safety, training and supervision should emphasize the importance of properly cleaning the patient’s finger, keeping lancets clean, and correctly disposing of used lancets. On some occasions in this study, CHWs were observed to excessively scrub a patient’s finger, place the lancet down after opening it, and placing used lancets and pipettes down after using them in the test procedure. Cleaning a patient’s finger is a necessary step in the procedure, but excessive scrubbing can result in residual quantities of alcohol on a patient’s finger that can impact test results. Once a lancet is opened, it should be held by the healthcare worker until it is properly disposed of in a sharps box. Placing the lancet down prior to use can jeopardize the sterility of the tool, potentially exposing the patient to infectious agents in the environment. Placing a lancet down following testing can result in a potentially unsafe situation where a sharp object that may be contaminated with blood could pose a risk to the CHW or other individuals in the testing vicinity. These actions have not been noted in other CHW studies but could compromise the safety and accuracy of the tests.

The accuracy of the test can be affected by incorrect blood volumes and timing of the test interpretation. In this study, blood collection techniques had improved at follow-up observations. However, observers noted that CHWs collected too little or too much blood on several occasions. Inadequate blood volumes can reduce test sensitivity, while excessive blood volumes can result in staining and obscuring test lines [36]. As opposed to other studies that have noted difficulties with blood collection apparatus [25, 28], the most frequently observed error in this study was inadequate amounts of blood. Researchers have also raised concerns about puncturing technique affecting blood volumes, noting that CHWs may set the lancet on the patient’s fingertip and attempt to push it in, rather than using a stabbing motion [28]. These actions were not observed in the assessments conducted in this study. Another error that was observed, although rarely, was reading RDT results early. This can compromise the accuracy of the test results. Other studies have also noted that reading RDT results early is a frequently observed error [27, 28] and have suggested time-saving motivations as one explanation for this practice [27].

The results showed that experience using RDTs were found to be significantly associated with lower error rates at follow-up observations. Other work has demonstrated that practicing RDT procedures is associated with improved performance [28]. The current study may have also reinforced previous trainings by building on existing skills.

Follow-up cross-sectional analysis and longitudinal analysis of errors rates at both time-points demonstrated that total errors and safety errors were higher for CHWs 50 years of age or greater compared to those younger than 40 years. Taken together, these results support the assertion that, on average, younger CHWs perform RDTs better than those over 50 years of age. The reasons for this are unknown, but potential explanations may be related to physical differences between these populations (e.g., vision), older CHWs attending a greater variety of training and using practices learned elsewhere that do not concur with the practices endorsed in this study, or differences in comfort and facility with new technologies. Other work has shown similar results. Counihan and colleagues [25] found that CHWs 50 years of age and older were less likely to perform as well as CHWs of 40 years or under for some indicators.

CHWs performed excellently when asked to interpret test results, achieving at least a sensitivity of 92% and a specificity of 97%. These results are similar to those reported in most studies that use microscopy as a gold standard for diagnosis [13]. Previous studies have also noted difficulties in the interpretation of faint-positive test lines by CHWs [24, 25, 28, 37], which was thought to be due to age-related vision degradation [24, 37]. While sensitivity was lower for cassettes with faint-positive lines, there was no observed effect of age on RDT test interpretation in this study. An important association was observed between having used a Deki reader and better interpretation, suggesting that real-time feedback on interpretation may improve their ability to read RDTs. Although this finding did not reach statistical significance in this study, future work with larger sample sizes should continue to investigate the potential of this technology to improve RDT techniques.

It is also worth noting that adherence to RDT test results is crucial for achieving positive health outcomes. Ultimately, even if CHWs perform RDTs at high or acceptable levels over time, the expected reductions in malaria morbidity and mortality will only be realized should patients follow treatment protocols. Gathering information regarding treatment adherence following RDT testing by CHWs would be required to fully endorse a community case management policy. Although outside the aims of this study, this could represent one avenue for future research.

This study had several notable strengths including a relatively large sample size for a study involving CHWs, a longitudinal study design, and employing the same observers at both time-points to standardize scoring. However, limitations do exist that may have influenced study results. Perhaps most significant of these concerns is missing baseline data for 13 of the CHWs. These missing data resulted from irretrievable baseline checklists. Although the sensitivity analyses conducted to compare the various populations found the sub-set of CHWs with missing data did not differ significantly from the rest of the study population, these data may have influenced the study findings or introduced biases based on unmeasured characteristics. Second, the presence of observers may have impacted CHW performance. Researchers have discussed how an awareness of being observed may affect the behaviour of study participants. Some suggest that formal observation can lead to a state of anxiety that holds negative consequences for behaviour [38]. Other works note increases in performance during supervision and evaluation [39]. In this study, the presence of observers may have impacted CHW performance in either fashion leading to an anxious state adversely affecting performance, or an increased adherence to preferred testing practices due to an awareness of the evaluation. It is not possible to predict what impact observers may have had on the results of this study [39]. Finally, previous work has noted that the maintenance of testing skills may also be related to regular supervision [25, 27]. CHWs met with study staff once per month, although the RDT procedure was not practiced or observed at these supervision meetings. Although meetings may have influenced morale and performance in a general manner, they likely did not impact the basic skills. This study cannot definitively distinguish the influence of training from supervision conducted by study staff.

## Conclusions

This work demonstrates that with proper training, most CHWs maintain their competency at conducting RDTs and interpreting subsequent results over a 12-month timeframe. Error rates are associated with some characteristics such as gender, age and prior experience with RDTs. Future programmes that engage CHWs to conduct RDTs should focus additional supervision and support on older CHWs, should encourage that testing be performed in environments conducive to accurately interpreting faint-positive test results, and emphasize the importance of explaining test results to patients. Additional research should be conducted to investigate the method by which CHW characteristics influence error rates in the RDT procedure.

## Additional files

**Additional file 1.** Information on missing CHW baseline data. Information pertaining to missing baseline data.

**Additional file 2.** Errors observed at follow-up observations. A list of the steps and corresponding errors observed at follow-up (12 month) observations.

## Abbreviations

ACT: artemisinin-combination therapy
CHEW: community health extension worker
CHW: community health worker
DR: Deki reader
GEE: generalized estimating equations
MER: mean error ratio
MOH: Ministry of Health
RDT: malaria rapid diagnostic test
