# Supplementary material for: Evaluation of malaria rapid diagnostic test (RDT) use by community health workers: a longitudinal study in western Kenya
Source: Malar J. 2018 May 18;17:206. doi: 10.1186/s12936-018-2358-6 (PMC5960182; doi:10.1186/s12936-018-2358-6)
Supplement: Supplementary file 1 — Additional file 1. Information on missing CHW baseline data. Information pertaining to missing baseline data. [file 12936_2018_2358_MOESM1_ESM.docx]

**Additional File I. Information on Missing Data**

| Baseline Checklist | Number of Observations (*n*) |  | Follow-Up Checklist | Number of Observations  (*n*) |
| --- | --- | --- | --- | --- |
| Step 1 | 90 |  | Step 1 | 103 |
| Step 2 | 90 |  | Step 2 | 103 |
| Step 3 | 90 |  | Step 3 | 103 |
| Step 4 | 90 |  | Step 4 | 103 |
| Step 5 | 90 |  | Step 5 | 103 |
| Step 6 | 90 |  | Step 6 | 103 |
| Step 7 | 90 |  | Step 7 | 103 |
| Step 8 | 90 |  | Step 8 | 103 |
| Step 9 | 90 |  | Step 9 | 103 |
| Step 10 | 90 |  | Step 10 | 103 |
| Step 11 | 90 |  | Step 11 | 103 |
| Step 12 | 88 |  | Step 12 | 103 |
| Step 13 | 90 |  | Step 13 | 103 |
| Step 14 | 89 |  | Step 14 | 103 |
| Step 15 | 86 |  | Step 15 | 103 |
| Step 16 | 90 |  | Step 16 | 103 |
| Step 17 | 89 |  | Step 17 | 103 |
| Step 18 | 87 |  | Step 18 | 103 |
| Step 19 | 83 |  | Step 19 | 103 |
| Step 20 | 83 |  | Step 20 | 103 |
