# Supplementary material for: Evaluation of malaria rapid diagnostic test (RDT) use by community health workers: a longitudinal study in western Kenya
Source: Malar J. 2018 May 18;17:206. doi: 10.1186/s12936-018-2358-6 (PMC5960182; doi:10.1186/s12936-018-2358-6)
Supplement: Supplementary file 2 — Additional file 2. Errors observed at follow-up observations. A list of the steps and corresponding errors observed at follow-up (12 month) observations. [file 12936_2018_2358_MOESM2_ESM.docx]

**Additional File 2. Errors Observed at Follow-Up Observations**

| **No.** | **Task** | **Errors Observed** |
| --- | --- | --- |
| 1. | Assembles necessary materials | - Forgot materials (lancet, pipette, buffer, swab, cotton) |
| 2. | Read RDT expiration date | - Did not check expiration date |
| 3. | Remove contents of test packet | - Opened packet with teeth |
| 4. | Write patient's name on cassette | - Did not write patient’s name on cassette |
| 5. | Identify patient’s details and date on the RDT cassette | - Did not write date on cassette |
| 6. | Explain procedure to patient | - Did not specify that they were testing for malaria  - Did not explain procedure to patient |
| 7. | Wear gloves | **-** Attempted to conduct RDT without wearing gloves |
| 8. | Select 4^th^ finger from the thumb of the left hand for blood collection | - Selected the wrong finger  - Selected the wrong hand |
| 9. | Clean finger with alcohol swab and allow it to dry | - Excessive wiping  - Cleaned with cotton instead of alcohol |
| 10. | Prick finger firmly with sterile lancet | - Placed lancet down after uncapping |
| 11. | Discard lancet in sharps bin immediately after pricking finger | - Placed used lancet on mat  - Placed used lancet in waste bag (non-sharps waste container) |
| 12. | Do not squeeze finger excessively | - Squeezed finger excessively to obtain blood sample |
| 13. | Collect an adequate volume of blood with pipette | - Did not wipe away first drop of blood  - Collected too little blood  - Collected too much blood |
| 14. | Dispense blood in correct well | - Dispensed collected blood in buffer well |
| 15. | Discards the pipette in the sharps box | - Placed used pipette in waste bag (non-sharps waste container) |
| 16. | Dispose of gloves & cotton wool in non-sharps container | - Did not remove used gloves  - Unintentionally smeared blood on other item (buffer, pen, phone, mat) |
| 17. | Dispense correct volume of buffer | - Dispensed too much buffer  - Missed buffer well while trying to dispense |
| 18. | Wait for twenty (20) minutes | - Attempted to read results too soon  - Did not set timer |
| 19. | Read results correctly | - Misinterpreted test result (faint-positive) |
| 20. | Verify internal test control | - Did not verify control strip |
